# Interpretation of within-group change in randomised trials

**DOI:** 10.1186/s12888-020-02641-w

**Published:** 2020-05-14

**Authors:** Kypros Kypri

**Affiliations:** Centre for Clinical Epidemiology and Biostatistics, School of Medicine and Public Health, University of Newcastle, Callaghan, New South Wales Australia

**Keywords:** Trial, Randomisation, Regression to the mean, Treatment, Inference, Equipoise

## Abstract

In medicine, it is common to observe improvement after intervention, at least partly because patients present for care in extremis and would have improved without intervention. Controlling for this counterfactual explanation for improvement is the principle reason to conduct a trial in which patients are randomised to treatment or a control group. Accordingly, it is not reasonable to infer that both interventions are effective when the groups show similar improvements in outcome.

## Main text

Säfsten and colleagues report a superiority trial comparing two counselling models delivered via a national alcohol helpline [1]. Their efforts are laudable given the need for effective countermeasures to the heavy burden of alcohol in Sweden and globally [2]. I offer comment regarding a fundamental inference they make from the study that the authors may reconsider.

In the trial registry (ISRCTN13160878) the investigators pre-specified the primary outcome as “Change of alcohol drinking habits measured with AUDIT score … [at] 6 and 12 month follow-up”, and in a subsequent published protocol, as “change from a higher to a lower AUDIT risk-level category between baseline and follow-up” [3].

Their finding was that among participants who provided follow-up data six months after randomisation, 61% of those allocated to receive usual care (reactive telephone counselling) versus 68% of those allocated to receive a novel intervention (less labour intensive telephone counselling with proactive elements), had AUDIT scores that placed them in the ‘low-risk drinking’ category [1]. Effect estimates expressed as a risk ratio (RR = 1.12; 95% CI: 0.93, 1.37), and a risk difference (RD = 0.08, 95%CI: − 0.05, 0.20), were judged as “[not showing] clear superiority for either counselling model”.

The authors present an open and nuanced discussion of the findings, however, in the conclusion of the main text (which is notably different from that in the abstract) they claim:

“A brief structured intervention did achieve favourable changes in problematic alcohol use … similar to those of a more labour intensive MI-based telephone counselling” [1] (p.8)

In addition to drawing an inference that extends beyond what a superiority trial can support, Säfsten and colleagues appear to have overlooked the simplest explanation for the “significant changes in clients’ AUDIT risk levels” [1] (p.8) they observed, namely, regression to the mean [4].

In research involving measurement of individuals at two or more points in time, such as typically occurs in a trial, it is common to see fluctuation in the outcome of interest, reflecting the natural history of the condition and/or measurement error [5].

Where people are screened-in to a study (e.g., by scoring ≥ 8 on the AUDIT), their scores will, on average, be lower upon later measurement. This is an arithmetic consequence of excluding from the trial people who score below the cut-off, whose scores, on average, would have increased if they had been measured later, offsetting the decreases in the group above the cut-off [4].

In their discussion of the null finding, Säfsten and colleagues make the astute observation that:

“ … many clients calling the [alcohol helpline] are likely to be highly motivated to change their behaviour, and probably already started the process of change before the first contact” [1] (p.7)

The tendency for people to seek help in extremis, when the course of a condition is at or near its peak and would probably improve without intervention, complicates inferences from uncontrolled observation. Lacking a counterfactual, clinicians are prone to over-estimate the effectiveness of some treatments [6]. For example, population-based studies showing that middle-ear infection typically remits without treatment (e.g., [7]) led to trials and then guidelines designed to reduce the over-prescription of antibiotics [8].

In a statistical demonstration of regression to the mean in alcohol research, colleagues and I analysed data from a cohort with a high prevalence of hazardous drinkers, finding that among people who scored ≥ 8 on the AUDIT at baseline, approximately half of the change in their scores at 6-month follow-up was attributable to regression to the mean [9]. Our motive for that study was the apparent tendency of researchers in the brief interventions and alcohol treatment fields to interpret reductions in drinking or harm that were not clearly greater in intervention groups than in comparator or control groups, as evidence that the conditions were equally effective [9].

Such an inference defies the logic of the randomised trial whose explanatory power depends on testing for differences in outcome between groups that were equivalent before the intervention of interest [10]. The protection against measured and unmeasured confounding achieved through randomisation of a sufficient number of individuals encompasses the artefact of regression to the mean because it occurs in both groups [10].

In the present case, proportional or absolute differences in the change in alcohol risk status, beyond those attributable to measured and unmeasured confounders, and regression to the mean, represent unbiased estimates of the superiority of the novel intervention over usual care. This is not to say that the alcohol helpline interventions studied here are ineffective, merely that this trial [1] does not speak to effectiveness per se.

Säfsten and colleagues assert that in the context of people calling a helpline, “a no-treatment control condition was considered unethical” [1] (p.8). However, in the absence of effectiveness data, equipoise is the only reasonable starting point [11]. Given scarce resources for the prevention and treatment of alcohol problems, it would be worth considering how one might design research to estimate the effects of an alcohol helpline versus the alternative of no such service.

## Data Availability

Not applicable.

## Abbreviations

AUDIT: Alcohol Use Disorders Identification Test
MI: Motivational Interviewing
RD: Risk Difference
RR: Risk Ratio
